# Metabolite Profiling Reveals a Specific Response in Tomato to Predaceous *Chrysoperla carnea* Larvae and Herbivore(s)-Predator Interactions with the Generalist Pests *Tetranychus urticae* and *Myzus persicae*

**DOI:** 10.3389/fpls.2016.01256

**Published:** 2016-08-25

**Authors:** Audrey Errard, Christian Ulrichs, Stefan Kühne, Inga Mewis, Narantuya Mishig, Ronald Maul, Mario Drungowski, Pia Parolin, Monika Schreiner, Susanne Baldermann

**Affiliations:** ^1^Leibniz Institute of Vegetable and Ornamental CropsGroßbeeren, Germany; ^2^Institute of Nutritional Science, University of PotsdamNuthetal, Germany; ^3^Urban Plant Ecophysiology, Faculty of Life Sciences, Humboldt-Universität zu BerlinBerlin, Germany; ^4^Julius Kühn-Institut, Federal Research Center for Cultivated Plants, Institute for Strategies and Technology AssessmentKleinmachnow, Germany; ^5^Julius Kühn-Institut, Federal Research Center for Cultivated Plants, Institute for Ecological Chemistry, Plant Analysis and Stored Product ProtectionBerlin, Germany; ^6^Hamburg School of Food Science, Institute of Food Chemistry, University of HamburgHamburg, Germany; ^7^Institut Sophia Agrobiotech, UMR 1355-7254, Institut National de la Recherche Agronomique-Center National de la Recherche Scientifique-Université de Nice Sophia AntipolisSophia Antipolis, France

**Keywords:** carotenoids, plant volatiles, Chrysopidae, Solanaceae, multiple-pest infestation, tritrophic system, Twister™, biological pest control

## Abstract

The spider mite *Tetranychus urticae* Koch and the aphid *Myzus persicae* (Sulzer) both infest a number of economically significant crops, including tomato (*Solanum lycopersicum*). Although used for decades to control pests, the impact of green lacewing larvae *Chrysoperla carnea* (Stephens) on plant biochemistry was not investigated. Here, we used profiling methods and targeted analyses to explore the impact of the predator and herbivore(s)-predator interactions on tomato biochemistry. Each pest and pest-predator combination induced a characteristic metabolite signature in the leaf and the fruit thus, the plant exhibited a systemic response. The treatments had a stronger impact on non-volatile metabolites including abscisic acid and amino acids in the leaves in comparison with the fruits. In contrast, the various biotic factors had a greater impact on the carotenoids in the fruits. We identified volatiles such as myrcene and α-terpinene which were induced by pest-predator interactions but not by single species, and we demonstrated the involvement of the phytohormone abscisic acid in tritrophic interactions for the first time. More importantly, *C. carnea* larvae alone impacted the plant metabolome, but the predator did not appear to elicit particular defense pathways on its own. Since the presence of both *C. carnea* larvae and pest individuals elicited volatiles which were shown to contribute to plant defense, *C. carnea* larvae could therefore contribute to the reduction of pest infestation, not only by its preying activity, but also by priming responses to generalist herbivores such as *T. urticae* and *M. persicae*. On the other hand, the use of *C. carnea* larvae alone did not impact carotenoids thus, was not prejudicial to the fruit quality. The present piece of research highlights the specific impact of predator and tritrophic interactions with green lacewing larvae, spider mites, and aphids on different components of the tomato primary and secondary metabolism for the first time, and provides cues for further in-depth studies aiming to integrate entomological approaches and plant biochemistry.

## Introduction

Since the spider mite *Tetranychus urticae* Koch and the aphid *Myzus persicae* (Sulzer) are highly fecund and quickly complete their life-cycle under similar climatic conditions (Van Emden et al., 1969; Wermelinger et al., 1991), they both infest a number of economically significant crops, including tomato (*Solanum lycopersicum*). The performance of *T. urticae* and *M. persicae* differs, depending on whether they are present separately or together on host-plants (Errard et al., 2015a), while the plant's metabolome is differently impacted by single- and multiple-pest infestation (Errard et al., 2015b). To date, a wide array of volatile and non-volatile compounds either constitutively present or induced in response to herbivory has been discovered. These biochemical defenses can promote plant tolerance and resistance, for example by repelling the pest (Schaller, 2008; Dicke et al., 2009). In general, pest infestation tends to up-regulate the secondary metabolism of the host-plant, and down-regulates its primary metabolism (Stam et al., 2014). However, both the nature and the role of the metabolites involved in the plant response appear to be specific to a particular pest-plant system (Agrawal, 2000; Zhou S. et al., 2015).

Since the chemical control of both *T. urticae* and *M. persicae* is compromised by their ability to develop pesticide resistance (Van Leeuwen et al., 2010; Bass et al., 2014), there is a need to promote and develop biological control methods. The larva of green lacewing *Chrysoperla carnea* (Stephens) has long been recognized as a predator of both *T. urticae* and *M. persicae*. In the context of tritrophic interactions, particular attention was paid to volatiles involved in indirect plant defense. Certain terpenoids can act as an attractant for Chrysopidae species, but the response of the predator may be dose-dependent. For instance, *C. carnea* adults were lured into field traps by baiting with two grams of β-caryophyllene (Flint et al., 1979), but a smaller amount (100 mg) proved to be ineffective (Zhu et al., 2005). On the other hand, as reviewed by Price et al. (1980), some of these volatiles have a negative impact on the natural enemies of the pest. As a result, the net benefit to the plant of this mode of defense remains debatable (Schaller, 2008). Recent studies exclusively focused on the population dynamics of arthropod pests in response to the presence of natural enemies and/or one selected class of plant metabolites responsive to the treatments (Messelink et al., 2014; Pappas et al., 2015). Here, we used profiling methods and targeted analyses to explore the overall impact of *T. urticae* and/or *M. persicae* in the presence/absence of *C. carnea* larvae on the biochemistry of tomato plants. The life cycle of *M. persicae* and *T. urticae* is completed within 1–3 weeks, and the latter's egg usually hatch within 1 week (Van Emden et al., 1969; Sabelis, 1981). Thus, under natural conditions, there is a delay of a few weeks between the occurrence of pest individuals in the crops and an infestation substantial enough to have any impact on crop yield and/or quality. The plant response to herbivory is strongly influenced by the length of the period of infestation (Zhou S. et al., 2015), and mainly the short term effects (from few hours to a few days after infestation) were monitored (reviewed by Schaller, 2008). In the present study the biochemical analyses were conducted after nearly 4 weeks of treatments. The carotenoids (tetraterpenes) were analyzed since they are important determinants of fruit quality and in human nutrition due to their anti-carcinogenic and anti-oxidative properties (Britton et al., 2008). Moreover, carotenoids and their volatile breakdown products can mediate plant-insect interactions (Schaefer and Rolshausen, 2006; Zheng et al., 2010; Heath et al., 2013; Cáceres et al., 2016). Given that carotenoids (9Z)-violaxanthin and (9Z)-neoxanthin are precursors for the synthesis of the phytohormone abscisic acid (ABA; Taylor et al., 2000), the ABA content of tomato leaves and fruits was also investigated. Finally, a targeted analysis of the free amino acids of tomato leaves and fruits was also performed. Amino acids are key compounds for the plant's primary metabolism and some accumulate in response to a wide range of abiotic and biotic factors such as γ-aminobutyric acid (GABA) and proline (Kinnersley and Turano, 2000; Szabados and Savouré, 2010).

## Materials and methods

### Plants, pests, and predators

Tomato plants *S. lycopersicum* var. “Ailsa Craig” (7 weeks-old plants) were grown under glasshouse conditions [22 ± 3°C; 40–70% relative humidity (RH); 14 h light/10 h dark]. The arthropods, *T. urticae*, and *M. persicae* were obtained from Katzbiotech AG (Baruth, Germany). The former species was reared on beans (*Phaseolus vulgaris* L. “Saxa”) and the latter on pak choi (*Brassica rapa* var. *chinensis* “Black Behi”). The predators *C. carnea* synchronized (±1 day) L2 larvae and both pests were reared for several generations under glasshouse conditions (25 ± 5°C, 55 ± 15% RH, photoperiod 16:8 h (light:dark).

### Experimental design and sampling method

To prevent the movement of either the two pests and of *C. carnea* larvae between plants, each plant was isolated within a cage made of transparent, micro-perforated material (Baumann Saatzuchtbedarf, Waldenburg, Germany). The tomato replicates were placed following a randomized sampling-design in the glasshouses. The treatments were maintained for 26 days and are hereafter coded: TU—infestation by *T. urticae* alone; MP—infestation by *M. persicae* alone; TUMP—simultaneous infestation by *M. persicae* and *T. urticae*; Predator—release of *C. carnea* larvae alone; TU-Predator—infestation by *T. urticae* in the presence of *C. carnea*; MP-Predator—infestation by *M. persicae* in the presence of *C. carnea*; and TUMP-Predator—simultaneous infestation by *M. persicae* and *T. urticae* in the presence of *C. carnea* (Table 1). The pest individuals used had reached the adult stage. The *C. carnea* larvae were released 10 days after the pest infestation. No effort was made to maintain the larvae on tomatoes. As a positive control for the jasmonate-mediated plant response, a 2.5 mM solution of methyl jasmonate [(MeJA), 0.1% v/v ethanol, 20 μL of Tween 20] was sprayed onto plants so that each received 16 ± 0.1 mg MeJA (MeJA treatment; Table 1). Shortly after the collection of emitted volatiles, a pool of fully-expanded leaves (10 ± 2 g fresh weight) was harvested per plant, as well as the fruits from each of the seven tomato plants. The material was freeze-dried in liquid nitrogen and stored at −80°C, then lyophilized and ground to a fine powder.

**Table 1:** **Time frame and treatments of tomato *S. lycopersicum* “Ailsa Craig” (7 weeks-old) with pest(s) and/or predator**.

	**Time frame**	**Day 1**	**Day 10**	**Day 24**	**Day 26**
	**Actions**	**Infestation (number of individuals/plant)**	**Addition of predators (number of larvae/plant)**		**Sampling**
*N*	Tomato treatments				
7	Control				1/Collection of volatiles 2/Collection of leaf and fruit material
7	TU (*T. urticae*)	200			
7	MP (*M. persicae*)	200			
7	TUMP (both)	100 of each			
7	Predator (*C. carnea*)		5		
7	TU-Predator	200	5		
7	MP-Predator	200	5		
7	TUMP-Predator	100 of each	5		
7	MeJA	spray (2.5 mM)		2.5 mM	

### Chemicals

The following chemicals and reagents were used for the analyses: methanol (99.95%), acetonitrile (99.99%), and ammonium acetate, purchased from Carl Roth GmbH and Co. KG (Germany); tetrahydrofuran (99.7%) from VWR International GmbH (Germany); methyl tert-butyl ether (99.8%) from Geyer GmbH & Co. KG (Germany); formic acid (98-100%), dichloromethane (99.9%), isopropanol (99.95%), Tween 20 from Serva Electrophoresis GmbH (Germany); methyl salicylate (MeSA) and methyl jasmonate (MeJA) from Merck AG (Germany); C7-C40 alkanes (Supelco-49452-U), terpinenes (α, δ, ɣ), trans-β-ocimene, *n*-hexanal, nonanal, nerolidol, β-pinene, myrcene, sabinene, phellandrenes (α, β), β-caryophyllene and β-carotene from Sigma-Aldrich Chemie GmbH (Germany); α-carotene, (9Z)-neoxanthin, zeaxanthin, violaxanthin from CaroteNature GmbH (Switzerland). Lutein was isolated from *Tagetes erecta* by flash chromatography. After saponification, the lutein was purified and crystallized. Its structure was elucidated by NMR and high-resolution mass spectrometry (Baldermann, 2008), and compared to the authentic reference compound purchased from Sigma-Aldrich Chemie GmbH (Germany).

### Analysis of metabolites, volatiles, and carotenoids

Five pairs of stir bar sorptive extraction devices (Gerstel-Twisters™, Polydimethylsiloxane phase, obtained from Gerstel GmbH & Co.KG, Germany) were used to simultaneously collect volatiles emitted from the adaxial and abaxial leaf epidermis of fully expanded leaves (see Errard et al., 2015b). Volatiles were collected from the full set of plants over a 20 min period in the early-morning, shortly before the harvesting of leaves and fruits. The volatiles were analyzed by GC-MS following Errard et al. (2015b), adjusting the oven temperature regime: 40°C over 3 min, rising by 2°C/min up to 60°C, held at 60°C for 2 min, then increased by 3°C/min up to 180°C, and finally held for 10 min isothermally. The compounds were identified tentatively by comparing the mass spectra with the Wiley 6.L and NIST 98.L libraries. The identification of the volatiles was confirmed using authentic compounds, when possible. The non-volatile metabolites were analyzed with a 1290 Infinity UHPLC coupled with an Agilent 6230 ToF LC-MS (Agilent Technologies GmbH, Germany) following Errard et al. (2015b), with minor modifications of the chromatographic gradient (A, 0.01% v/v aqueous formic acid; B 0.01% v/v formic acid in acetonitrile): B was increased from 2 to 5% over 3 min, from 5 to 15% over 10 min, from 15 to 80% over 8 min and finally to 100% over 2 min. The flow rate was 0.4 mL/min. An electrospray ionization (ESI) source was used and the spectra were collected in both positive and negative ionization mode over a 70–1200 *m/z* range (capillary voltage, 3.5 kV; source temperature, 320°C; nebulizer gas flow, 8 L/min at 35 psi). The data were converted and processed by Mass Profiler Professional (MPP; Version 12.1, Agilent Technologies; USA) following Errard et al. (2015b), adjusting the parameters for the recursive workflow step to generate compound formulae (match tolerance of 10 ppm). Briefly, after peak-picking, alignment of the detected features, integration, and peak area calculation, MPP enables multivariate data analyses such as principal component analysis (PCA) to determine and visualize the dispersion between two or more sample groups and variables. Each sample was normalized to the median of the baseline and log 2 transformed. A one-way ANOVA (*p* ≤ 0.01; fold change ≥ 2) was performed to identify the different features impacted significantly. The metabolites significantly impacted by the treatments were tentatively identified using Mass Hunter Metlin PCD (version 4.0, 24768 compounds) and in-house databases (see Errard et al., 2015b). The analysis of carotenoids was performed following Errard et al. (2015b). Briefly, the carotenoids were extracted three times from 10 mg of lyophilized samples using 0.5 mL of MeOH/THF solution (1:1, v/v). The extracts were mixed (1000 rpm, 5 min, room temperature), centrifuged (4000 g, 5 min, 20°C), evaporated in a stream of nitrogen, and dissolved in 0.02 mL of dichloromethane and 0.18 mL of isopropanol before filtration through a 0.2 μm polytetrafluoroethylene membrane. The carotenoids were separated on a YMC C30 column (100 × 2.1 mm, 3 μm, YMC Co. Ltd., Japan) and analyzed by UHPLC-ToF-LCMS using an APCI ion source in positive ionization mode (Agilent Technologies). The data analysis was performed by using Mass Hunter ToF Quantitative Analysis (version B 05.00, Agilent Technologies) following Errard et al. (2015b).

### ABA quantification

Each step of the extraction was performed under darkness and on ice. ABA was extracted five times from a 10 mg sample of finely powdered, lyophilized fruit or leaf material with 200 μL methanol solution (MeOH 60%, formic acid 0.01% v/v). It was then sonicated 15 min and centrifuged (4500 g, 7 min, 4°C). Volumes were adjusted to 1 mL with the methanol solution. After 1 h incubation at 4°C, the extracts were filtered through a 0.2 μm polytetrafluoroethylene membrane. Stock solutions (MeOH 60%) with (+)-ABA were prepared (0.05, 0.1, 0.15 μg/mL). For quantification, applying the standard addition method, four aliquots (50 μL) from each sample were prepared, adding 50 μL of MeOH 60% or (+)-ABA from the different stock solutions. Volumes were adjusted to 500 μL with ultra-pure water. The samples were analyzed by liquid chromatography (HPLC Agilent 1260 Infinity, Agilent Technologies, USA) coupled to a triple quadrupole, Q-Trap® 6500 ESI-MS/MS system (Sciex, USA). The samples (injection volume, 4 μL) were separated using an Ascentis Express F5 column (15 cm × 4.6 mm, 5 μm) (Supelco, Sigma-Aldrich Co. LLC, USA). Samples and column temperature were kept at 4° and 35°C, respectively. The chromatographic gradient was composed of two solutions (solvent A, 0.3% v/v aqueous formic acid; solvent B 0.3% v/v formic acid in acetonitrile) at a flow rate of 0.65 mL/min. A was maintained at 90% over 1 min, decreased to 65% over 6 min, decreased to 10% over 2 min, maintained at 10% over 1 min, increased to 90% over 0.85 min and maintained at 90% over 3 min. The ESI interface was used in negative-ionization mode at 400°C with the following settings: curtain gas, 40 psi; nebulizer gas, 50 psi; auxiliary gas, 60 psi; ionization voltage, −4500 V; collision gas setting high declustering potential, −80 V; entrance potential, −10 V; cell exit potential, −9 V; selected-reaction-monitoring (SRM) dwell time, 25 ms. SRM transitions monitored for ABA were 263.0 → 153.0 [quantifier, collision energy (CE), 20 V] and 263.0 → 219.0 (qualifier, CE 15V). Data acquisition and integration were achieved using Analyst 1.6.2 software (Sciex, USA). Microsoft Excel 2010 was used for the calculations of linear regressions.

### Analysis of amino acids

Each step of the extraction was performed under darkness and on ice. The amino acids were extracted from a 10 mg sample of finely powdered, lyophilized fruit or leaf material once in 250 μL 70% methanol (pH 2, kept at 4°C) followed by 15 min sonication on ice and centrifugation (4000 g, 5 min, 4°C). The amino acids were then extracted twice with 100 μL 70% methanol (pH 2, kept at 4°C), followed by a 10 min sonication on ice and centrifugation (4000 g, 5 min, 4°C). Volumes were adjusted to 400 μL to which was added 100 μL precipitation solution (MembraPure GmbH, Germany). After 1 h incubation at 4°C, the extracts were filtered through a 0.22 μm cellulose acetate membrane. The amino acids analysis was performed using a MembraPure Amino Acid Analyzer (MembraPure GmbH, Hennigsdorf, Germany) following manufacturer's instructions. The data were analyzed with the Chromatography Data Handling System (Amino peak v. 2.36, MembraPure GmbH, Germany).

### Statistical analyses

The statistical analysis of volatiles, carotenoids, ABA, and amino acids was performed using SPSS 21 (one-way ANOVA, Tukey's HSD *post-hoc* test, *p* ≤ 0.05).

### Pathway visualization

The results of the targeted and non-targeted analyses were combined to illustrate the interplay of pest infestations and pest-predator interactions on the biochemistry of tomato plant (**Figure 5**). The pathway map (**Figure 5**) was created after Errard et al. (2015b), based on the Kyoto Encyclopedia of Genes and Genomes (KEGG; maps 01230, 00250, 01060, 00900, and 01062), and using ChemDraw Std (version 13.0) to draw the chemical structures of the compounds.

## Results

### Non-volatile metabolite profiling

**Figure 1:**
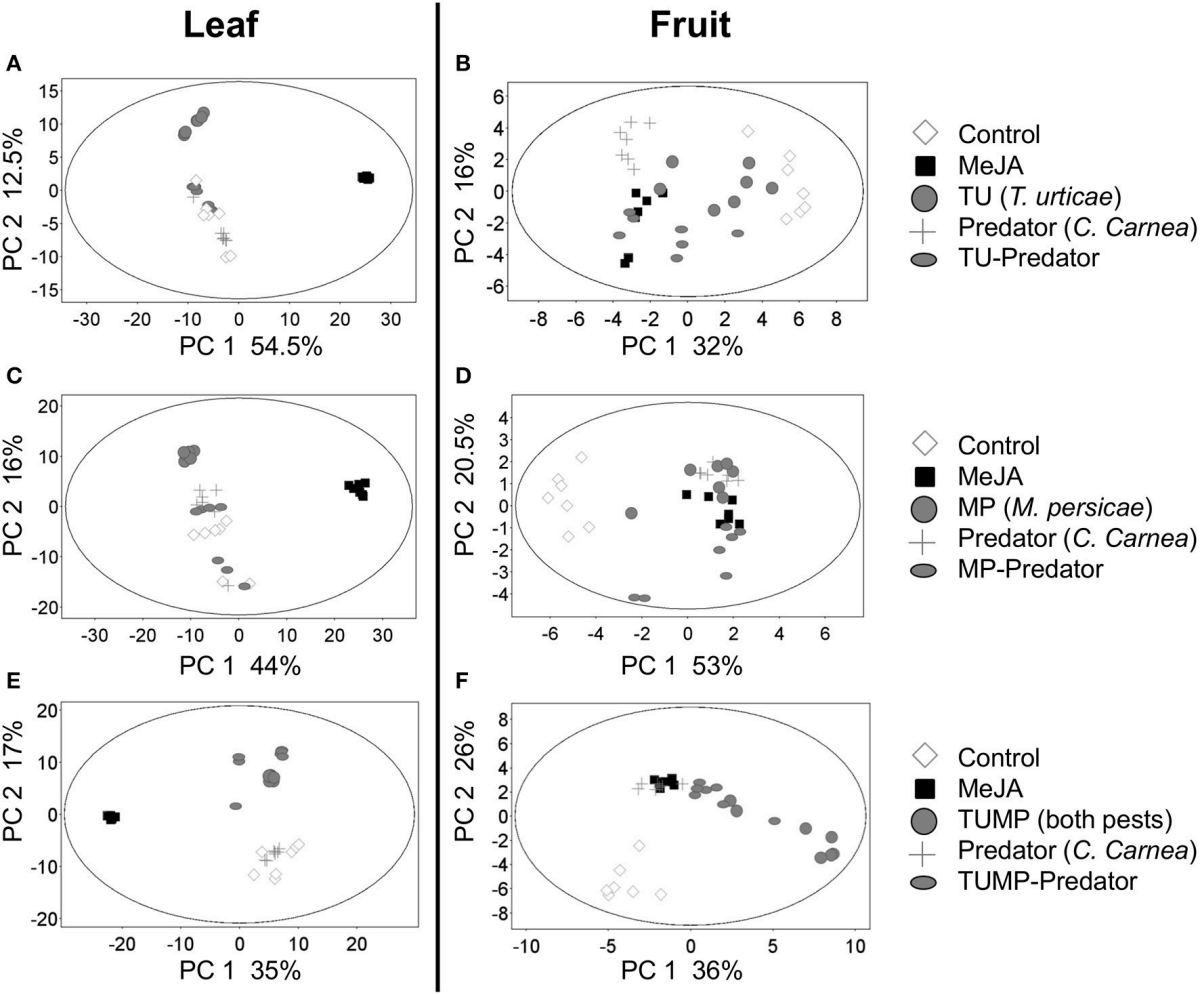
**Principal component (PC) analyses (*p* ≤ 0.01, fold change ≥ 2) of the metabolites tentatively identified [M–H]^−^ 70 to 1200 *m/z* range (Supplemental Table S1) in (A,C,E) leaves and (B,D,F) fruits of tomato *S. lycopersicum* “Ailsa Craig” after 4 weeks of treatment (*N* = 7) with spider mites *T. urticae* (TU) and/or aphids *M. persicae* (MP), in the presence or absence of *C. carnea* larvae (Predator); MeJA, plants sprayed with methyl jasmonate, 2.5 mM**.

A diversity of treatment-induced metabolites was identified, including secondary metabolites such as fatty acids, polyphenols, phenylpropanoids, isoprenoids, and plant hormones related to specific pathways such as salicylate and jasmonate. Primary metabolites such as sugars and compounds related to the citrate cycle were also identified. Metabolites involved in the amino acid metabolism were particularly conspicuous (Supplemental Table S1) thus, a targeted analysis of amino acids was subsequently performed to assess their concentration in tomato tissues (see Free Amino Acids section). The principal component analyses (Figure 1) revealed that the global set of plant metabolites impacted by the Predator treatment differed from those elicited either by TU or TU-Predator (Figures 1A,B), MP or MP-Predator (Figures 1C,D), TUMP or TUMP-Predator (Figures 1E,F). These results indicate that the plant response to pest-predator combinations was not simply a cumulative one compared to the impact of single species. Therefore, the interaction between pest(s) and the predator induced a specific metabolite composition in *S. lycopersicum*. Since the impact of the treatments could be observed in both the leaf and the fruit, the plant exhibited a systemic response to the different treatments. Furthermore, the Predator treatment elicited a response that differed from control plants, especially in fruit, demonstrating that the presence of *C. carnea* larvae in the absence of either pest was not neutral on tomato biochemistry. A comparison of the biotic treatments with the MeJA treatment identified distinct clusters of leaf metabolites (Figures 1A,C,E), suggesting that the jasmonic acid pathway was little or not induced by the biotic factors, or that other hormonal pathways were elicited.

### Volatile profiling

The blend of volatiles impacted by the treatments and emitted from the upper epidermis was composed of 20% monoterpenes, 40% sesquiterpenes, 27% aldehydes, 7% alkanes, and 7% fatty acid derivatives. From the lower epidermis, the blend of volatiles impacted by the treatments was composed of 37% monoterpenes, 32% sesquiterpenes, 5% aldehydes, 11% phenylpropanoids, and 5% fatty acid derivatives. These results show that the emission of volatiles differed between the abaxial and adaxial leaf epidermis (Table 2), supporting a previous study (Errard et al., 2015b). The volatiles with the highest abundance were phellandrene isomers and β-caryophyllene, but they were not significantly impacted by the treatments (Supplemental Table S3). From the adaxial epiderme, the predator elicited the emission of volatiles such as δ-terpinene, but did not significantly differ from control plants. The monoterpene myrcene, which was emitted from leaves treated with both spider mites and predators (TU-Predator) was not emitted from either TU- nor Predator-treated plants. From the abaxial epiderme, α-terpinene was not emitted in response to either the predators alone or the aphids, but it was emitted by the MP-predator treatment. In contrast, the sesquiterpenes germacrene D, δ-cadinene, β-maalienne, and γ-elemene which were emitted in response to spider mites, were not detected in the TU-Predator treatment. The TUMP-Predator treatment induced a specific emission of β-maalienne from the abaxial epiderme, and β-elemene from the abaxial apiderme. Of note, neither the monoterpenes α- and δ-terpinene nor the sesquiterpenes β-maalienne and β-elemene were emitted from MeJA-treated plants. These results suggest that other phytohormonal pathways were elicited by the different biotic factors, and support the findings of the non-volatile profiling.

**Table 2:** **Mean abundance of the volatiles emitted from tomato *S. lycopersicum* “Ailsa Craig” leaves which were significantly impacted after 4 weeks of treatment with spider mites *T. urticae* and/or aphids *M. persicae* in the presence/absence of predaceous *C. carnea* larvae**.

**Epiderme**	**Class**	**Compound**	**RT**	**Treatments [Mean Peak Area ±*SD* (10^6^)]**								
				**Control**	**MeJA**	**Predator**	**TU**	**TU-predator**	**MP**	**MP-predator**	**TUMP**	**TUMP-predator**
adaxial	fatty acids	methyl jasmonate^*^	41.5	0a	57.81 ± 61.27b	0a	0a	0a	0a	0a	0a	0a
	alkanes	nonadecane^*^	47.6	6.45 ± 5.68a	44.89 ± 16.57b	0a	0a	0a	17.50 ± 3.49a	44.47 ± 19.03b	0a	0a
	aldehydes	*n*-hexanal^*^	6.1	0a	0a	0a	13.38 ± 10.08b	12.72 ± 5.41b	0a	0a	16.77 ± 6.46b	0a
		nonanal^*^	22.8	17.02 ± 5.74ab	37.41 ± 12.79a	0b	20.37 ± 9.95ab	16.67 ± 5.78ab	15.03 ± 3.98ab	34.34 ± 30.05a	14.04 ± 6.04ab	20.12 ± 7.18ab
		decanal	27.2	13.32 ± 6.60ab	19.31 ± 9.89b	10.03 ± 2.58ab	0a	0a	15.74 ± 10.47b	17.03 ± 6.78b	7.54 ± 2.27ab	13.59 ± 6.93ab
	monoterpenes	myrcene^*^	16.7	0a	41.31 ± 37.15b	0a	0a	21.65 ± 14.50b	0a	0a	0a	0a
		allo-ocimene	24.1	4.09 ± 3.31a	0a	0a	0a	0a	0a	0a	27.09 ± 14.46b	8.14 ± 0.93a
		δ-terpinene^*^	22.1	17.21 ± 5.48ab	0a	19.22 ± 9.85ab	8.10 ± 5.80ab	23.31 ± 17.05b	17.79 ± 6.06ab	24.48 ± 13.91b	0a	0a
	sesquiterpenes	γ-elemene	35.4	2.79 ± 1.41ab	4.07 ± 4.04ab	0a	0a	5.67 ± 3.05ab	0a	6.74 ± 5.94b	0a	0a
		δ-elemene	32.3	61.79 ± 27.09ab	98.04 ± 49.03ab	40.73 ± 20.06ab	92.42 ± 55.68ab	70.88 ± 21.68ab	128.72 ± 119.90a	0b	57.66 ± 44.39ab	70.24 ± 30.98ab
		germacrene D	37.0	9.46 ± 6.32a	10.87 ± 6.56a	0b	0b	0b	0b	10.40 ± 1.77a	7.87 ± 4.64ab	8.74 ± 5.98ab
		β-maalienne	37.3	0a	0a	0a	0a	0a	0a	0a	0a	15.61 ± 9.32b
		nerolidol^*^	39.3	4.71 ± 2.72a	6.42 ± 4.77a	0b	0b	0b	0b	0b	0b	0b
		3,7-guaiadiene	35.8	0a	0a	0a	13.59 ± 7.39b	0a	10.92 ± 4.06b	13.57 ± 8.41b	11.92 ± 1.23b	9.35 ± 1.67ab
abaxial	fatty acids	methyl jasmonate^*^	41.5	0a	6.22 ± 4.63b	0a	0a	0a	0a	0a	0a	0a
	phenyl-propanoids	methyl salicylate^*^	26.6	1.89 ± 1.59ab	0a	0a	0a	0a	0a	6.13 ± 10.30ab	9.33 ± 1.60b	8.39 ± 2.94ab
		eugenol^*^	32.6	1.77 ± 1.43a	0b	1.61 ± 0.95ab	0b	0b	0b	0b	2.41 ± 1.11a	0b
	alkanes	cyclododecane	36.7	4.91 ± 3.48a	0b	0b	0b	0b	0b	0b	0b	0b
		cyclohexadecane	47.3	26.73 ± 29.91a	0b	0b	0b	0b	0b	0b	0b	0b
	aldehydes	5-methyl fufural	14.1	5.35 ± 1.52b	0a	0a	0a	0a	0a	0a	0a	0a
	monoterpenes	β-pinene^*^	15.6	5.97 ± 3.12a	0a	6.62 ± 5.17a	11.54 ± 9.21b	0a	0a	0a	0a	0a
		*trans*-iso-limonene	17	16.78 ± 10.09a	0b	13.16 ± 12.80ab	22.18 ± 8.78a	0b	0b	0b	17.92 ± 9.33a	0b
		myrcene^*^	16.7	7.66 ± 3.97a	0b	0b	0b	18.87 ± 6.46a	0b	0b	0b	0b
		sabinene^*^	15.3	3.60 ± 1.69b	0a	0a	0a	0a	0a	0a	0a	0a
		*trans*-β-ocimene^*^	20.1	3.88 ± 2.09a	0b	3.64 ± 2.80a	4.67 ± 4.69b	0b	0b	0b	0b	0b
		α-terpinene^*^	18.3	4.57 ± 2.54ab	0a	0a	5.70 ± 3.38ab	0a	0a	16.36 ± 19.41b	0a	0a
		γ-terpinene^*^	20.6	6.36 ± 3.34a	0b	0b	0b	0b	0b	0b	0b	0b
	sesquiterpenes	nerolidol^*^	39.3	1.33 ± 0.51a	0b	0b	0b	0b	0b	0b	0b	0b
		germacrene D	37	0a	0a	0a	9.81 ± 6.77b	0a	10.98 ± 7.21b	0a	0a	0a
		δ-cadinene	37.8	0a	0a	0a	8.71 ± 5.02b	0a	4.36 ± 6.45ab	0a	0a	0a
		β-maalienne	37.3	0a	0a	0a	10.67 ± 7.24b	0a	0a	0a	0a	0a
		β-elemene	34.1	3.53 ± 3.02a	0a	0a	10.02 ± 8.66a	0a	0a	0a	0a	74.30 ± 49.47b
		γ-elemene	35.4	1.84 ± 0.93ab	0a	0a	3.86 ± 3.16b	0a	0a	0a	0a	0a

*MeJA, elicitation with methyl jasmonate (2.5 mM); MP, aphids M. persicae; TU, spider mites T. urticae; Predator, green lacewing larvae C. carnea. RT, retention time (min)*.

* *Identification confirmed by co-injection with authentic compounds*.

*Different lower case letters indicate significant differences between the treatments (p ≤ 0.05, one-way ANOVA, Tukey's HSD post-hoc test, N = 4)*.

### Carotenoids

**Figure 2:**
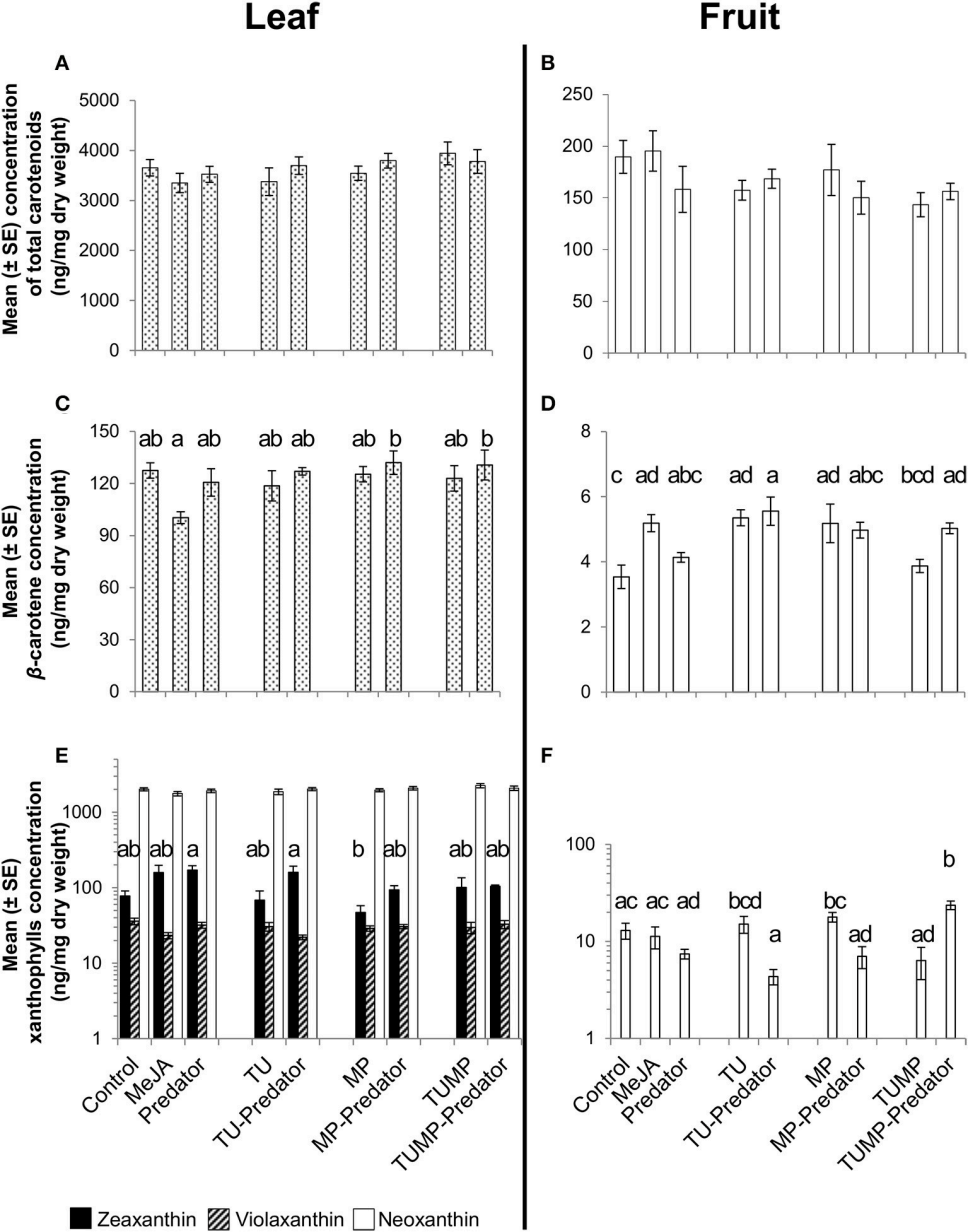
**Carotenoid content in leaves (A,C,E) leaves and (B,D,F) fruits of tomato *S. lycopersicum* “Ailsa Craig” after 4 weeks of treatment with spider mites *T. urticae* (TU) and/or aphids *M. persicae* (MP), in the presence or absence of *C. carnea* larvae (Predator); MeJA, plants sprayed with methyl jasmonate, 2.5 mM**. Different lower case letters indicate significant differences (one-way ANOVA, Tukey' HSD *post-hoc* test, *p* ≤ 0.05; leaves *N* = 7; fruits *N* = 6).

The total carotenoid content of both the leaves and fruits was not markedly influenced by the treatments (Figures 2A,B). β-Carotene is the precursor of the xanthophylls zeaxanthin, neoxanthin, and violaxanthin. On the basis of single carotenoids, the concentration of β-carotene was not significantly impacted by the treatments in the leaves in comparison to control plants (Figure 2C). In the fruits, there was significantly more β-carotene in plants exposed to the MeJA, TU, TU-Predator, MP and TUMP-Predator treatments compared to control plants (Figure 2D). The treatments did not impact xanthophylls in the leaves in comparison to control plants (Figure 2E). Neither violaxanthin nor zeaxanthin were detectable in the fruit (Figure 2F), but neoxanthin was differently impacted by the treatments. In comparison to pests alone (TU and MP treatments), the single pest-predator combinations (TU-Predator and MP-Predator treatments, respectively) decreased the neoxanthin concentration in the fruits. A multiple infestation in the presence of the predator (TUMP-Predator) resulted in higher neoxanthin content in the fruits in comparison with control plants. Predators alone did not impact single carotenoids. However, the results also indicate that pest and pest-predator interactions could influence the xanthophylls in tomato plants. Given that violaxanthin and neoxanthin are precursors for the synthesis of ABA, the ABA content in tomato leaves and fruits was also investigated.

### ABA

**Figure 3:**
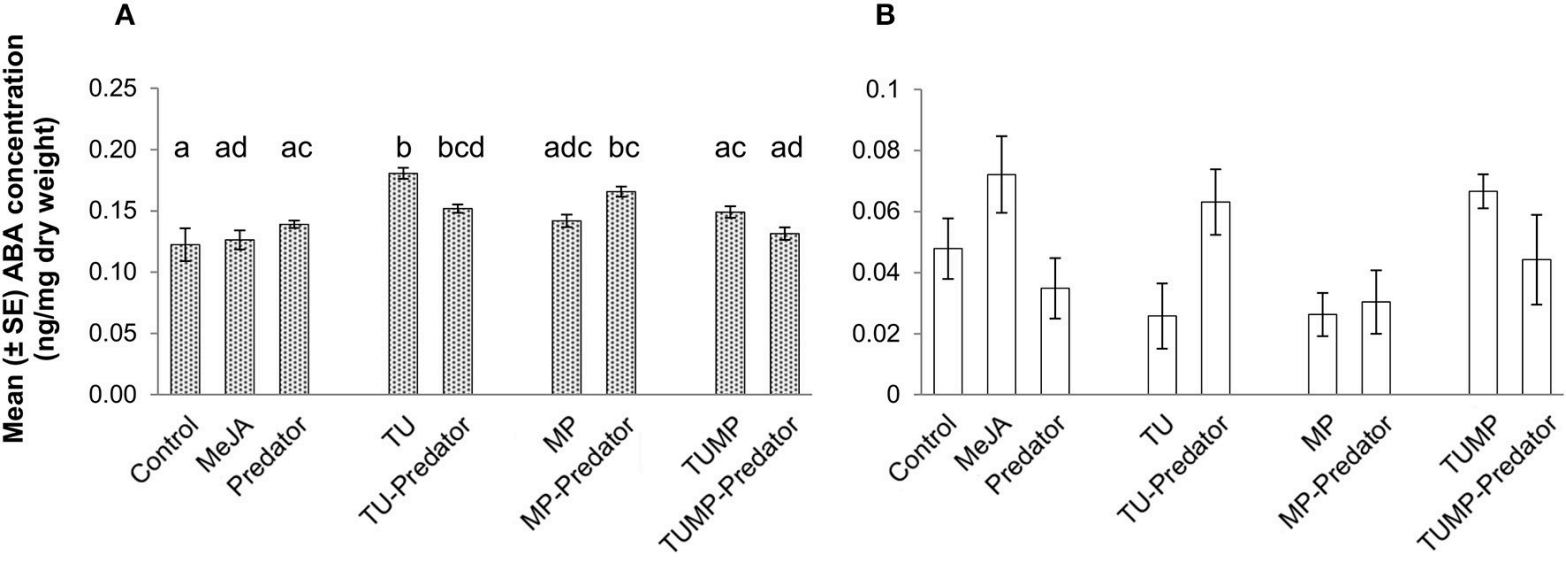
**Abscisic acid concentration in (A) leaves and (B) fruits of tomato *S. lycopersicum* “Ailsa Craig” after 4 weeks of treatment with *T. urticae* (TU) and/or aphids *M. persicae* (MP), in the presence or absence of *C. carnea* larvae (Predator); MeJA, plants sprayed with methyl jasmonate, 2.5 mM**. Different lower case letters indicate significant differences (one way ANOVA, Tukey' HSD *post-hoc* test, *p* ≤ 0.05; leaves *N* = 7; fruits *N* = 6).

In comparison to control plants, the presence of the predator alone did not impact the concentration of ABA. The leaf ABA concentration was higher in response to the TU and TU-Predator treatments compared to control plants (*p* ≤ 0.05; Figure 3A). Contrary to aphids alone (MP), the MP-Predator treatment resulted in higher ABA content in the leaves. The presence of both the pests with and without predators (TUMP and TUMP-Predator treatments) did not significantly impact the leaf ABA content, suggesting that the presence of several species induced a phytohormonal crosstalk in tomato plants. The fruit ABA concentration was not significantly impacted by any treatment (Figure 3B).

### Free amino acids

**Figure 4:**
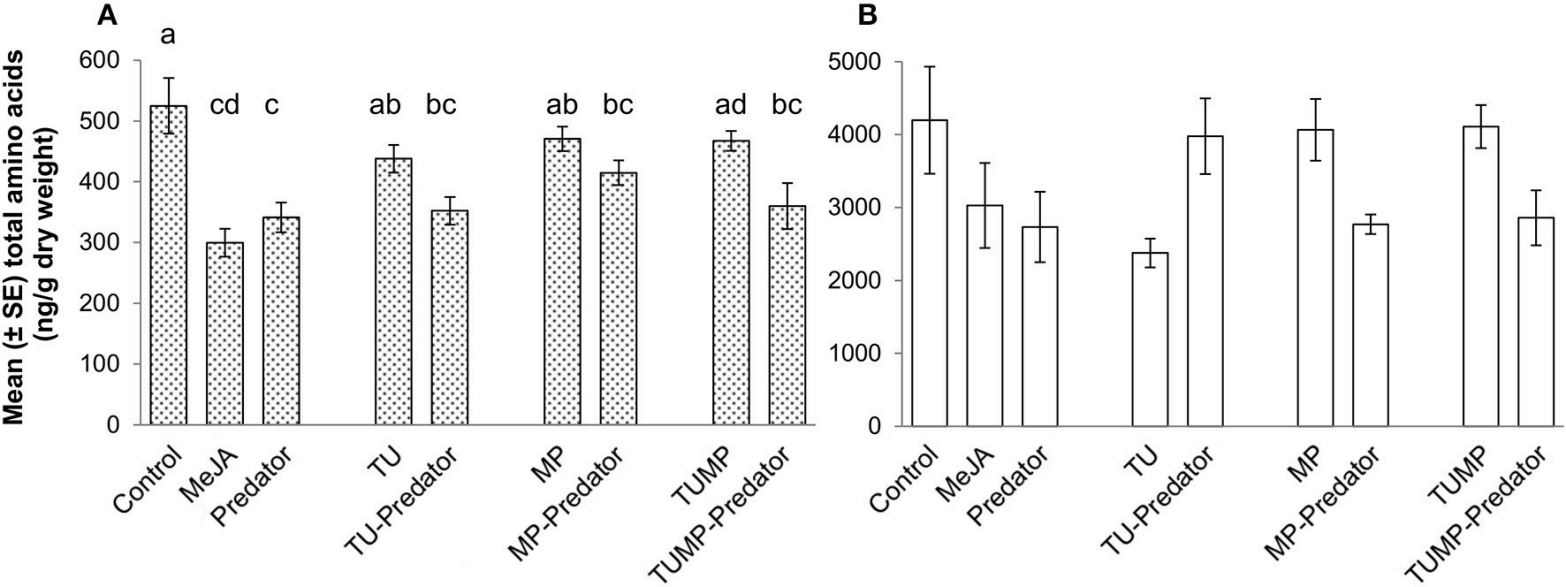
**Total free amino acids in (A) leaves and (B) fruits of tomato *S. lycopersicum* after 4 weeks of treatment with spider mites *T. urticae* (TU) and/or aphids *M. persicae* (MP), in the presence or absence of *C. carnea* larvae (Predator); MeJA, plants sprayed with methyl jasmonate, 2.5 mM**. Different lower case letters indicate significant differences (one-way ANOVA, Tukey' HSD *post-hoc* test, *p* ≤ 0.05; leaves *N* = 7; fruits *N* = 6).

**Figure 5:**
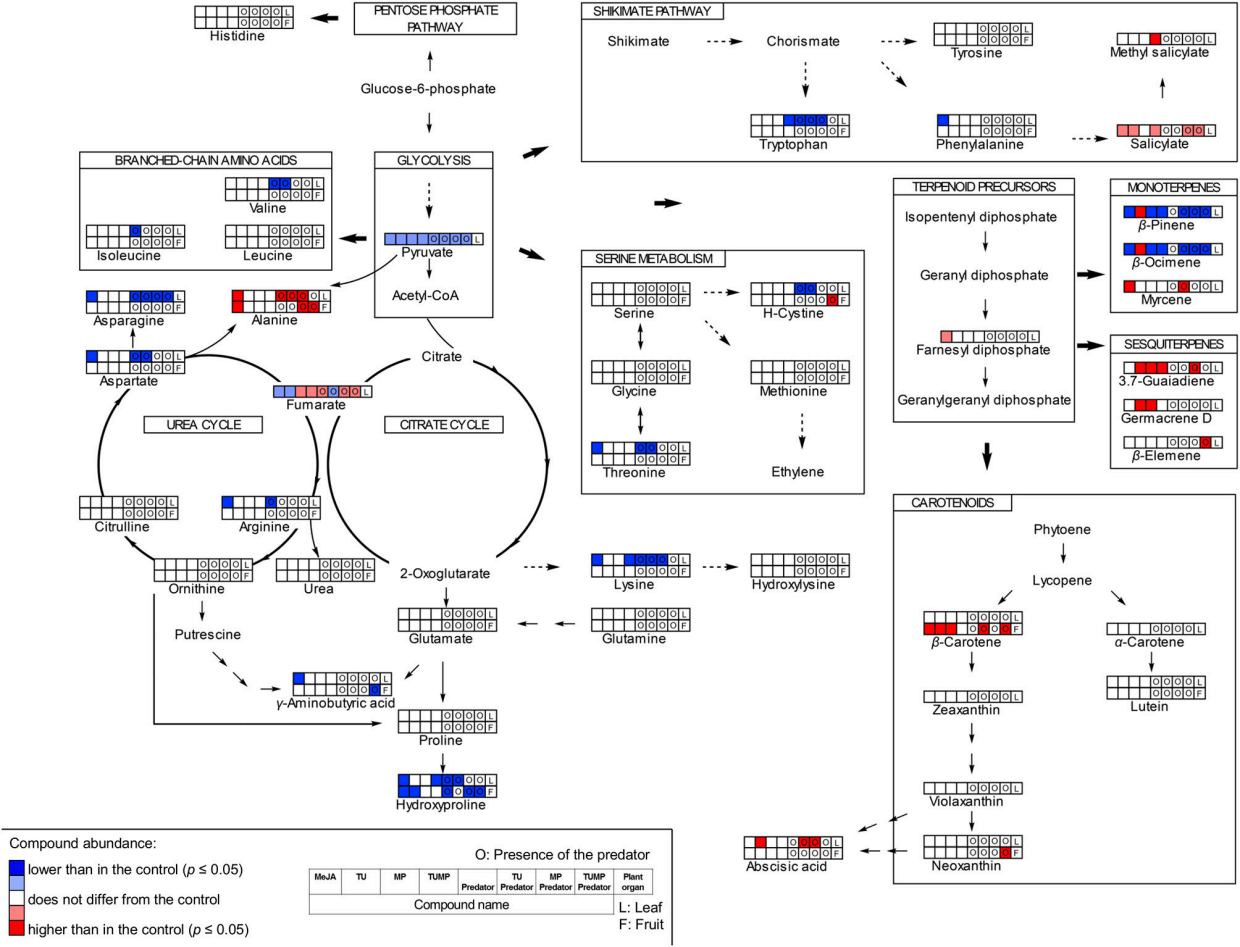
**A schematic of the pathways induced in the leaf and in the fruit of tomato *S. lycopersicum* “Ailsa Craig” by the pests *T. urticae* (TU) and *M. persicae* (MP) in the presence or absence of predaceous *C. carnea* larvae after a 4 weeks of treatment; MeJA, plants sprayed with methyl jasmonate, 2.5 mM (one-way ANOVA, Tukey' HSD *post-hoc* test, *p* ≤ 0.05; leaves *N* = 7; fruits *N* = 6)**.

The total free amino acid content in the leaf was significantly reduced in response to the MeJA, Predator, TU-Predator, MP-Predator, and TUMP-Predator treatments (Figure 4A). The total amino acid concentration in the fruits was not affected by the treatments. The single amino acids serine, glutamate, glutamine, leucine, glycine, methionine tyrosine, histidine, hydroxylysine, and proline were not impacted by the treatments (Supplemental Table S2). Together with tyrosine, tryptophan and phenylalanine are related to the shikimate pathway. The phenylalanine concentration was significantly lower only in response to the MeJA treatment in the leaves. Lower tryptophan content was detected in response to the Predator, TU-Predator, MP-Predator, and TUMP treatments. From the serine metabolism we could detect serine, glycine, methionine, threonine, and the oxidized form of cysteine (H-cystine). Threonine was not impacted in the fruits, but a lower concentration was found in response to the MeJA, Predator, and TU-Predator treatments in the leaves. Less H-cystine was induced in response to the TUMP-Predator treatments in the fruits, and in response to the Predator and TU-Predator treatments in the leaves. Valine, leucine, and isoleucine derive from pyruvate. These amino acids were not impacted in the fruits. In the leaves, the predator reduced the concentration of both valine and isoleucine. A lower valine concentration was also found in response to the TU-predator treatment. Urea, aspartate, asparagine, alanine, citrulline, ornithine, and arginine belong to the urea cycle (Figure 5), and aspartate is the precursor of both alanine and asparagine. In the leaves, the MeJA, Predator, and TU-predator treatments decreased the concentration of aspartate and asparagine, and increased the alanine concentration in comparison to control plants. The MP-Predator and TUMP-Predator treatments decreased the asparagine concentration and increased the alanine content. In the fruit, a higher alanine concentration was induced by the MeJA and MP-predator treatments. Both the elicitation with MeJA and the presence of the predator induced a lower arginine concentration in tomato leaves in comparison to control plants. Glutamate and glutamine derive from the citrate cycle. Both the urea and the citrate cycles intervene in the synthesis of GABA, proline, and hydroxyproline (Figure 5). A lower hydroxyproline concentration was induced in both the tomato leaves and fruits by the MeJA and Predator treatments. The MP-Predator and TUMP-Predator treatments induced a lower hydroxyproline level in the fruits, but not in the leaves. In contrast, the TU-predator and a multiple infestation (TUMP) reduced the leaf hydroxyproline concentration. The GABA concentration in the fruits was not impacted by the treatments in comparison to control plants, and an infestation with aphids (MP treatments) resulted in higher GABA accumulation in tomato leaves in comparison to most treatments (MeJA, Predator, TU, MP-predator, and TUMP-Predator). In the leaves, the MeJA treatments induced a lower GABA content.

## Discussion

### The impact of predaceous *C. carnea* larvae on tomato biochemistry

**Figure 6:**
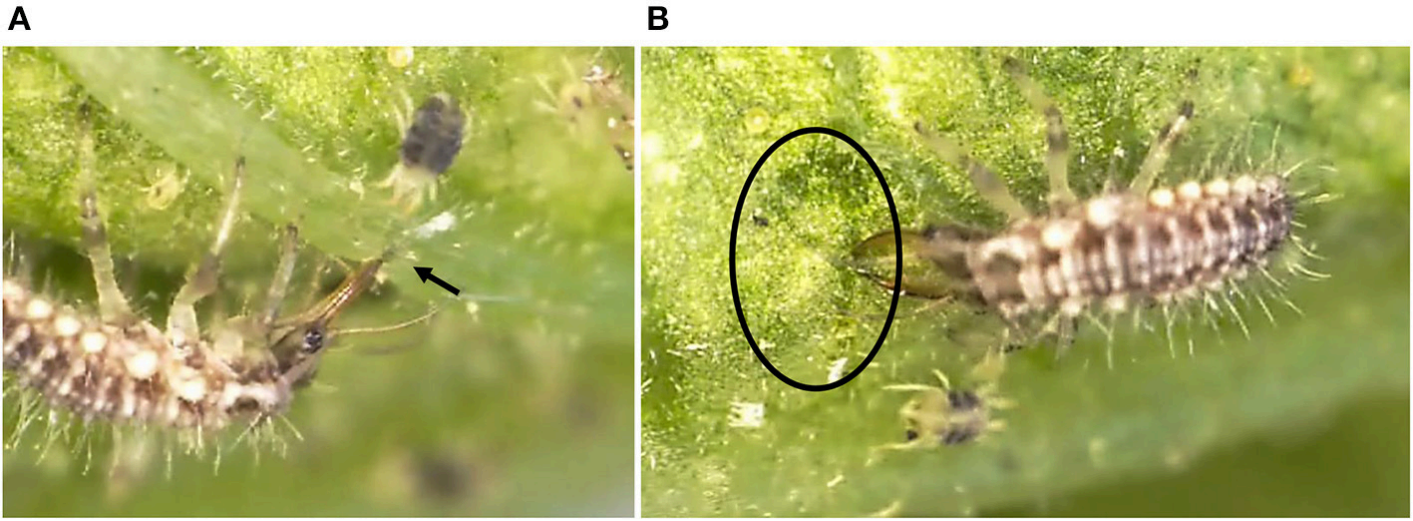
**(A)** *C. carnea* larva inserting its feeding part into the tomato, *S. lycopersicum* “Ailsa Craig” leaf vein axil (arrow), **(B)** Leaf area damaged by the *C. carnea* larva (circle; Digital microscope VHX-1000, Keyence; zoom × 10).

Although no *C. carnea* larvae were seen on plants exposed to the Predator treatment 10 days after their release, which may results from the onset of cannibalism brought on by food (prey) shortage (McEwen et al., 2001), the metabolite profile of the tomato plants differed when comparing the Predator treatment and control plants. Therefore, the predator can influence the plant chemical composition even in absence of arthropod pests (Figure 1). Larvae were observed to have inserted their piercing-sucking stylets into the leaf vein axil and in the leaf epidermis (Figure 6, Supplemental Video S4), presumably reflecting a survival behavior to access a source of carbohydrates (Villenave, 2006; Hogervorst et al., 2008). The resulting wounding damage, along with the possible presence of active compounds in the larval saliva, is highly likely to elicit a plant response including the emission of volatiles. Although some monoterpenes such as β-ocimene and δ-terpinene, and sesquiterpenes such as δ-elemene were present in the blend elicited by the predator, the emission of these compounds was not significantly different from the control. Literature mainly focused on the most abundant volatiles emitted by tomato leaves such as β-caryophyllene. The latter was shown to be involved in indirect plant defense by attracting *Chysoperla* species (Flint et al., 1979). In our study, the emission of β-caryophyllene was not impacted by the treatments (Supplemental Table S3). β-Caryophyllene may therefore play no major role in indirect plant defense, supporting a previous study (Zhu et al., 2005). Predators alone did not impact ABA and its precursors (xanthophylls) and a comparison of the Predator with the MeJA treatment identified distinct clusters of leaf metabolites (Figures 1A,C,E). Taken together, these results suggest that pathways other than those involving JA and ABA were elicited by the presence of predators. In general, the predator reduced the total amino acids in the leaves. The amino acid phenylalanine was not significantly impacted by the predator (Supplemental Table S2), no methyl salicylate (Table 2), and no salicylate (Supplemental Table S1) could be detected in response to the predator, suggesting that the predator alone does not impact the shikimic acid pathway. Alone, the predator treatment reduced the concentration of threonine, valine, and isoleucine. These amino acids can serve as precursors for volatiles defense compounds such as aldehydes (Dudareva et al., 2013). We also found a lower concentration of asparagine and its precursor aspartate in the Predator treatment. Apart from the feeding activity and wounding damage caused by the predator, there is increasing evidence showing the contribution of endosymbionts associated with arthropods to plant defense (Barr et al., 2010; Chung et al., 2013; Chaudhary et al., 2014). Recently, *Enterobacter* spp. and yeast symbionts associated with green lacewing adults and larvae could be identified (Woolfolk and Inglis, 2004; Hemalatha et al., 2014). Therefore, studying the possible implication of the microflora of *C. carnea* larvae on crops may offer novel insights into the interaction between this predator species and plant metabolism. Finally, the Predator treatment influenced neither the carotenoid synthesis (Figure 2) nor the total amino acid content in tomato fruits (Figure 4B). Therefore, the study supports that *C. carnea* larvae were not prejudicial to the fruit quality.

### The impact of multitrophic interactions on tomato biochemistry

The range of non-volatile metabolites elicited by the various pest-predator treatments demonstrated that the plant response was not a cumulative one to each species alone. Therefore, the plant response to the pest-predator combinations induced a specific metabolite composition in *S. lycopersicum*. From volatiles, aldehydes such as n-hexanal differed markedly between the TUMP and TUMP-Predator treatments (Table 2). It was also induced in response to spider mites in the presence and absence of predators (TU and TU-Predator treatments). The emission of hexanal in response to spider mite infestation was also reported in apple trees (Llusià and Peñuelas, 2001) and higher levels were recorded from tomato subjected to a multiple-infestation with spider mites and aphids (Errard et al., 2015b). In contrast, no hexanal was emitted in response to aphids (MP), supporting a previous study (Chehab et al., 2008) and the MP-Predator treatment. These results highlight the specificity of the tomato response to different pests. Particular monoterpenes were elicited in response to the pest-predator combinations but not by single species. For instance, α-terpinene was not emitted in response to either the predators alone or the aphids, but it was emitted in response to the MP-predator treatment. Terpinene isomers are known to be involved in plant response to generalist pests (Errard et al., 2015b), and contribute to plant defense by for instance, repelling pests such as whiteflies (Bleeker et al., 2009). Since the presence of both *C. carnea* larvae and pest individuals impacted both volatiles which can contribute to plant defense, this predator could therefore contribute to the reduction of pest infestation, not only by its preying activity on pests but also by influencing the plant biochemistry. Studying the effects of plant volatiles such as terpinene isomers on the biology of *C. carnea* using amounts comparable to those emitted by the plant would contribute to decipher the benefit of such chemical defense for plant fitness. Extensive literature is available on the elicitation of mono- and sesquiterpenes by pest including spider mites and aphids in the context of direct and indirect plant defense (Schaller, 2008; Dicke et al., 2009). The present study supports the importance of this class of compounds in the response of tomato plants to generalist pests such as spider mites and aphids, and for multitrophic interactions. Further work integrating entomology and biochemistry would enable to connect and/or correlate the elicitation of plant metabolites with pest population dynamics, and provide information on the efficiency of a preventive release of *C. carnea* larvae against pest infestations. In contrast to volatile terpenoids, the concentration of total carotenoids (tetraterpenes) was not affected by the treatments and no significant impact on the single carotenoids was detected in the leaves. However, variations in β-carotene and xanthophyll content were observed in tomato fruits (Figure 2). A higher β-carotene concentration was detected in MeJA-treated plants, TU, TU-Predator, MP, and TUMP-Predator treatments compared to control plants (Figure 2D). Lower neoxanthin was observed in response to single pest-predator combinations (TU-Predator and MP-Predator treatments) in comparison to pests alone (TU and MP, respectively), and the TUMP-Predator treatment prevented the reduction of neoxanthin in comparison with a multiple infestation with both spider mites and aphids (TUMP treatment). These results indicate that pest and pest-predator interaction could influence the xanthophylls in tomato plants. Since the isomerisation of the xanthophylls violaxanthin and neoxanthin can lead to the formation of ABA (Taylor et al., 2000; Zhou J. et al., 2015) a targeted analysis of this phytohormone was subsequently performed. The treatments did not impact the ABA concentration in the fruits. In the leaves, more ABA was synthetized in response to spider mites. A higher ABA concentration was previously reported in other tomato cultivars infested with the carmine spider mite *T. cinnabarinus* (Gawroñska and Kiełkiewicz, 1999). Together with previous findings, our study therefore suggests that spider mite species can elicit the ABA pathway in plants. Higher levels of ABA following aphid infestation were found in other plants such as *Medicago truncatula* and *Arabidopsis thaliana* (respectively, Guo et al., 2016; Hillwig et al., 2016). One effect of ABA signaling would lead to stomatal closure and thereby reduce leaf transpiration, allowing the aphids to modulate host tissue cell turgor, which is necessary for the continuity of their feeding activity (Huberty and Denno, 2004). However, here, the MP treatment did not detectably enhance plant ABA content. In contrast, more ABA was synthetized in the leaves subject to the aphid-predator combination (MP-Predator) and spider mites-predator combination (TU-Predator; Figure 3A). To our knowledge, this is the first study demonstrating the involvement of ABA in tritrophic interactions. Although spider mites alone induced a higher ABA concentration, no significant effects were observed in case of multiple-pest infestation with and without predators (TUMP and TUMP-Predator treatments; Figure 3). There is ample evidence that demonstrate phytohormonal crosstalk in plants subjected to multiple attackers (reviewed by Stam et al., 2014). It is likely that the feeding of both *T. urticae* and *M. persicae* on the same host in the presence or abscence of *C. carnea* interfered with the plant's hormone signaling network, leading to a specific blend of metabolites (Figure 1). To date, the role of ABA in the response to biotic factors, as opposed to abiotic ones, remains controversial and its effects are likely time-dependent (Ton et al., 2009). Moreover, the nature of a biotic factor has a major influence over the nature of the crosstalk occurring between the ABA and other phytohormone pathways (Ton et al., 2009; Nahar et al., 2012); such differences may well underlie the observed specificity of the tomato response to a particular combination of herbivore and predator. Bodenhausen and Reymond (2007) showed that in *A. thaliana*, feeding activity of pests such as *Pieris rapae* and *Spodoptera littoralis* feeding reprogrammed the transcription of a number of ABA-regulated genes involved in amino acid metabolism. We found that all treatments negatively impacted or tended to reduce total amount of amino acids (Figure 4). It has been proposed that the redirection of primary metabolites away from the organs being attacked can reduce their nutritive value to the pest, and thereby compromise the performance of the pests (Schaller, 2008; Zhou S. et al., 2015). Our results are consistent with this hypothesis. Some amino acids might also have served as precursors for the synthesis of particular defense pathways. For instance, it is possible that the shikimate pathway was also involved in the plant response to the aphid-predator combination (MP-predator treatment) since we observed the elicitation of salicylate in tomato leaves (Supplemental Table S1), emission of MeSA from the abaxial leaf epidermis (Table 2) and a reduction of the tryptophan content in the leaves (Supplemental Table S2). Further, studies are necessary to elucidate the involvement of the shikimic acid metabolism in the plant response to *Chrysoperla* species interacting with insect pests.

Although GABA accumulates in plants exposed to abiotic stress such as drought and to biotic factors especially virus and pathogens (reviewed by Kinnersley and Turano, 2000), we observed lower GABA content in the fruits subject to the TUMP-Predator treatment, and in the leaves elicited with MeJA (Supplemental Table S2). Since the plant's response to biotic stressors may depend on the duration of the infestations (Maeda and Takabayashi, 2001; Zhou S. et al., 2015), it is possible that the accumulation of these stress indicators occurs shortly after treatments. Further, studies comparing the short-term and long-term impact of tritrophic interactions would shed light on the metabolome dynamics.

In summary, our study highlighted the specific impact of different trophic levels on different components of the tomato primary and secondary metabolism (Figure 5). The profiling of non-volatile and volatile metabolites showed that the pest(s) and interactions between the pest(s) and the predator not only affected the regulation of endogenous non-volatile metabolites (Figure 1), but also induced a specific biochemical signature with regard to the blend of volatiles emitted by the plants (Table 2). The plant exhibited a systemic response, but the different treatments had a stronger impact on non-volatile metabolites, abscisic acid, and amino acids in the leaves, in comparison with the fruits. In contrast, the carotenoids were more impacted in the fruits. In general amino acids concentration decreased, supporting that infestation down-regulates the plant primary metabolism (Stam et al., 2014). In contrast, the responses of secondary metabolites in the tomato leaf and fruit depend to an extent on the identity of the pest(s) and/or-predator present. We showed the involvement of ABA in tritrophic interactions for the first time (TU-Predator and MP-Predator treatment) (Figure 3), but more investigations are necessary to understand the mechanisms which intervene in the plant response to multiple species (TUMP-Predator). *C. carnea* larvae significantly impacted the plant metabolome, but the predator alone did not appear to elicit particular defense pathways on its own. However, the presence of both *C. carnea* larvae and pest individuals elicited volatiles which were shown to contribute to plant defense. Therefore, C. carnea larvae could contribute to the reduction of pest infestation, not only by its preying activity, but also by priming responses to generalist herbivores. Finally, the presence of the predator was not prejudicial to the fruit quality. Future work combining the implications of our study and optimization of biological control methods for instance, by improving the installment and the sustainability of predator populations would contribute to fully benefit from these natural enemies.

## Author contributions

Conception and design of the work: AE, SK, CU, SB, IM, PP; Analytics: AE, SB, NM (metabolites, carotenoids, volatiles), AE (amino acids); AE, RM, SB (ABA); Figures: AE, NM, MD; Data interpretation: AE, NM, SB, CU, RM, SK, MS; Manuscript revision and approval: AE, NM, SB, CU, RM, SK, PP, IM, MD, MS; Accountability: AE, NM, SB, CU, RM, SK, PP, IM, MD, MS.

## Funding

The study was supported by IGZ which is funded by the Federal Ministry of Food and Agriculture, the Ministry of Sciences, Research and Cultural Affairs of Brandenburg and the Thuringian Ministry for Infrastructure and Agriculture. The work of AE was also supported by the German Academic Exchange Service (DAAD scholarship A/12/70428).

### Conflict of interest statement

The authors declare that the research was conducted in the absence of any commercial or financial relationships that could be construed as a potential conflict of interest.

## Supplementary material

Supplementary Table S1 **Tentative identification (±10 ppm) of tomato metabolites (*S. lycopersicum* “Ailsa Craig”) significantly impacted after four weeks of treatment with spider mites *T. urticae* and/or aphids *M. persicae* in the presence/absence of predaceous *C. carnea* larvae (*p* ≤ 0.01, one-way ANOVA, fold change ≥ 2, *N* = 7)**.

Supplementary Table S2 **Targeted-analysis of free amino acids. (A)** Mean (±*SD*) concentration of free amino acids in the leaves of tomato *S. lycopersicum* “Ailsa Craig” after 4 weeks of treatment with spider mites *T. urticae* and/or aphids *M. persicae* in the presence/absence of predaceous *C. carnea* larvae (ng/g dry weight). **(B)** Mean (±*SD*) concentration of free amino acids in the fruits of tomato *S. lycopersicum* “Ailsa Craig” after 4 weeks of treatment with spider mites *T. urticae* and/or aphids *M. persicae* in the presence/absence of predaceous *C. carnea* larvae (ng/g dry weight).

Supplementary Table S3 **Mean (±*SD*) peak area of the most abundant volatiles emitted by tomato *S. lycopersicum* “Ailsa Craig” leaves after four weeks of treatment with spider mites *T. urticae* and/or aphids *M. persicae* in the presence/absence of predaceous *C. carnea* larvae**.

Supplemental Video S1 ***C. carnea* larva on a leaf of tomato *S. lycopersicum* “Ailsa Craig.”**.

## Abbreviations

ABA: abscisic acid
APCI: atmospheric pressure chemical ionization
CE: collision energy
ESI: electrospray ionization
GABA: γ-aminobutyric acid
GC-MS: gas chromatography-mass spectrometry
JA: jasmonic acid
MeJA: methyl jasmonate
MeOH/THF: methanol/tetrahydrofuran
MeSA: methyl salicylate
MP: *Myzus persicae*
NMR: nuclear magnetic resonance
PCA: principal component analysis
RH: relative humidity
SA: salicylic acid
SE: standard error of the mean
SRM: selected-reaction-monitoring
TU: *Tetranychus urticae*
UHPLC-ToF-MS: ultra-high performance liquid chromatography-time-of-flight-mass spectrometry.
