# Supplementary material for: Metabolite Profiling Reveals a Specific Response in Tomato to Predaceous Chrysoperla carnea Larvae and Herbivore(s)-Predator Interactions with the Generalist Pests Tetranychus urticae and Myzus persicae
Source: Front Plant Sci. 2016 Aug 25;7:1256. doi: 10.3389/fpls.2016.01256 (PMC4997045; doi:10.3389/fpls.2016.01256)
Supplement: Supplementary Table S1 — Tentative identification (±10 ppm) of tomato metabolites (S. lycopersicum “Ailsa Craig”) significantly impacted after four weeks of treatment with spider mites T. urticae and/or aphids M. persicae in the presence/absence of predaceous C. carnea larvae (p ≤ 0.01, one-way ANOVA, fold change ≥ 2, N = 7). [file Table1.PDF]

Supplemental Table S1 **Tentative identification (± 10 ppm) of tomato metabolites (*S. lycopersicum* \`Ailsa Craig´) significantly impacted after 4 weeks of treatment with spider mites *T. urticae* and/or aphids *M. persicae* in the presence/absence of predaceous *C. carnea* larvae ($p \leq 0.01$, one-way ANOVA, fold change ≥ 2, N = 7)**

				Mean peak area (Log2)	Treatments (Log2-Fold change compared to the Control)							
**ESI $^{+/-}$**	**Metabolism of:**	**Compound name**	**Organ**	**Control**	**MeJA**	**Predator**	**TU**	**TU-Predator**	**MP**	**MP-Predator**	**TUMP**	**TUMP-Predator**
-		2,3-dihydroxybenzoate	leaves	-0.72	-4.07	-0.64	1.29	-0.24	0.32	0.17	-0.69	0.68
-		2-oxo-3-phenylpropanoate	leaves	-3.46	-13.45	-1.39	-5.17	-7.74	1.23	-5.38	0.95	-3.24
+		2-oxo-3-phenylpropanoate	fruits	-0.51	0.23	0.08	0.00	0.00	0.52	-0.17	0.00	0.00
-		L-threonine	leaves	-11.44	0.37	-7.67	-7.62	-3.79	-9.60	-1.92	0.26	0.83
-		L-tryptophan	leaves	-0.15	1.02	0.12	-0.12	0.08	-0.11	-0.04	0.05	0.85
-		*n*-carbamoyl-L-aspartate	leaves	-11.03	-4.75	-5.37	-4.16	-10.96	2.35	-4.88	-11.09	-0.20
-		*n*-succinyl-L,L-2,6-diaminopimelate	leaves	-9.93	0.19	-1.96	-1.74	-0.13	-4.22	-0.06	0.17	0.98
-		*(S)*-methyl-5'-thioadenosine	leaves	-11.57	-11.57	-1.34	-8.25	-3.23	-4.84	0.34	0.46	-7.89
-		*β*-alanine	leaves	-11.44	0.37	-7.67	-7.62	-3.79	-9.60	-1.92	0.26	0.83
-	amino acids	glutarate or quinate	leaves	-0.54	-3.94	-0.41	1.78	-0.02	0.40	0.32	-0.78	-0.25
-		gentisate	leaves	-0.72	-4.07	-0.64	1.29	-0.24	0.32	0.17	-0.69	0.68
-		*n*-butanoate	leaves	0.00	14.20	0.00	0.00	0.00	0.00	0.00	0.00	0.00
+		4-hydroxybutanoate	fruits	-0.65	0.19	0.41	0.00	0.00	0.31	-0.04	0.00	0.00
-		O-succinyl-L-homoserine	leaves	0.00	14.85	0.00	0.00	0.00	0.00	0.00	3.87	8.65
-		2-amino-3-oxobutanoate	leaves	-8.04	-1.82	-8.21	0.64	-6.32	0.24	0.27	-0.80	0.37
-		tyramine	leaves	10.17	15.62	7.57	0.00	0.00	1.89	0.00	0.00	0.00
-		4-hydroxyphenylpyruvate	leaves	-4.26	-16.18	-4.55	-5.76	-8.87	1.03	-6.49	-0.01	-1.87
+		discadenine	fruits	-0.72	-0.03	0.30	0.00	0.00	0.14	0.03	0.00	0.00
-		succinate semialdehyde	fruits	-1.82	0.11	1.15	0.00	0.00	0.00	0.00	-14.90	-4.67
+		3-hydroxyphenyllactate	fruits	-0.51	0.23	0.08	0.00	0.00	0.52	-0.17	0.00	0.00
-		10*S*,11*R*-dichloro-7,11-dimethyl-3-methylene-4*R*-hydroxy-6*E*,8*E*,12-tridecatrienoic acid	fruits	-6.74	-16.70	-3.73	0.00	0.00	0.00	0.00	0.97	-6.85
-	isoprenoids	(2*E*,6*E*)-farnesyl diphosphate	leaves	0.00	14.40	0.00	0.00	0.00	0.00	0.00	0.00	0.00
-		(3*R*)-linalool	leaves	0.07	-13.88	0.09	0.08	0.00	0.14	-0.09	-2.66	-7.64
-		sinapyl-alcohol	leaves	3.39	9.97	0.00	0.00	1.89	0.00	0.00	12.72	11.01
-	phenylpropanoids	trans-caffeate	leaves	-4.26	-16.18	-4.55	-5.76	-8.87	1.03	-6.49	-0.01	-1.87
-		esculin	leaves	-0.72	-4.07	-0.64	1.29	-0.24	0.32	0.17	-0.69	0.68
-		4-coumarate	leaves	-3.46	-13.45	-1.39	-5.17	-7.74	1.23	-5.38	0.95	-3.24
-		salicylate	leaves	0.00	16.17	0.00	3.45	0.00	0.00	2.26	15.63	13.43
-	polyphenols	3'-O-methyl-(-)-epicatechin-7-O-sulphate	fruits	-12.90	0.96	0.41	-7.61	-1.54	-2.15	-3.10	0.00	0.00
-		(-)-11-hydroxy-9,10-dihydrojasmonic acid 11-*β*-D-glucoside	fruits	-6.02	0.93	0.32	-0.20	-6.01	0.00	0.00	0.00	0.00
-		(-)-jasmonate	leaves	0.00	14.20	0.00	0.00	0.00	0.00	0.00	0.00	0.00
-	fatty acids	(9*Z*)-12-oxo-dodec-9-enoate	leaves	0.15	-12.85	0.14	-1.78	0.02	-1.72	-1.83	-12.85	-8.79
-		(9*S*,10*E*,12*Z*)-9-hydroperoxy-10,12-octadecadienoate	leaves	0.07	1.11	-0.21	-0.08	-0.17	-0.39	-0.19	0.66	1.01
+		(*S*)-3-hydroxydodecanoic acid	fruits	-13.96	-3.90	0.22	0.00	0.00	-3.93	0.11	0.00	0.00
-	ascorbate and aldrate	D-glucarate	leaves	0.64	-16.07	0.41	-0.78	0.30	-0.17	0.07	-0.40	-1.98
-		XMP	leaves	0.00	13.45	0.00	0.00	0.00	0.00	1.74	7.10	13.74
-	purine and alkaloids	xanthine	fruits	-3.99	1.06	3.96	-10.62	-7.00	0.00	0.00	-13.30	-5.06
-		purine	fruits	2.02	6.47	16.35	10.56	1.97	0.00	0.00	-14.90	-4.67
-		8-hydroxypurine	fruits	-0.01	-7.53	0.76	0.29	-0.32	0.00	0.00	0.00	0.00
-	precursor citrate cycle and glycolysis	pyruvate	leaves	1.25	-15.64	0.88	-0.25	0.52	-0.10	-0.16	0.27	-2.38
-	glycolysis	D-erythrose 4-phosphate or phosphoenolpyruvate	leaves	-10.53	-14.77	-2.15	0.57	-0.14	0.46	0.32	-2.26	-4.86
-		stachyose	leaves	9.02	14.59	9.18	1.82	5.86	0.00	3.86	5.43	1.79
-		*α*-D-galactose 1-phosphate or *α*-D-glucose 6-phosphate	leaves	0.00	14.99	0.00	0.00	0.00	0.00	0.00	0.00	7.17
-	sugars	*α*-D-glucose	leaves	-0.07	-9.05	-0.05	-0.16	0.05	0.24	-0.12	-0.36	0.85
-		dolichyl *β*-D-glucosyl phosphate	fruits	-8.24	0.54	0.58	-5.22	-1.66	0.00	0.00	-3.42	-11.90
+		erythritol	fruits	-0.65	0.19	0.41	0.00	0.00	0.31	-0.04	0.00	0.00
-		citrate or 2-oxoglutarate	leaves	-2.79	-16.90	0.57	-0.65	0.41	-0.25	-0.20	0.40	0.10
-		fumarate	leaves	-4.66	-15.64	-2.19	-6.39	-6.53	0.46	-2.05	-0.01	-2.33
-	citrate cycle	succinate	leaves	-13.67	0.55	-1.99	-9.89	-1.79	-11.75	0.13	0.05	1.04
-		*cis*-aconitate or D-threo-isocitrate or L-dehydro-ascorbate	leaves	-1.88	1.14	-9.21	-8.53	-8.80	-2.22	-1.92	0.07	1.08
-		L-malic acid	fruits	-3.91	1.14	4.04	0.00	0.00	0.00	0.00	-13.30	-5.06

abbreviations: MeJA, elicitation with methyl jasmonate (2.5 mM); MP, aphids *M. persicae*; TU, spider mites *T. urticae;* Predator, green lacewing larvae *C. carnea*

zero value: the compound was not detected

compound classification after the Kyoto Encyclopedia of Genes and Genomes (KEGG) (www.genome.jp/kegg) and PubChem (www.ncbi.nlm.nih.gov/pccompound)
