# Supplementary material for: Metabolite Profiling Reveals a Specific Response in Tomato to Predaceous Chrysoperla carnea Larvae and Herbivore(s)-Predator Interactions with the Generalist Pests Tetranychus urticae and Myzus persicae
Source: Front Plant Sci. 2016 Aug 25;7:1256. doi: 10.3389/fpls.2016.01256 (PMC4997045; doi:10.3389/fpls.2016.01256)
Supplement: Supplementary Table S2 — Targeted-analysis of free amino acids. (A) Mean (±SD) concentration of free amino acids in the leaves of tomato S. lycopersicum “Ailsa Craig” after 4 weeks of treatment with spider mites T. urticae and/or aphids M. persicae in the presence/absence of predaceous C. carnea larvae (ng/g dry weight). (B) Mean (±SD) concentration of free amino acids in the fruits of tomato S. lycopersicum “Ailsa Craig” after 4 weeks of treatment with spider mites T. urticae and/or aphids M. persicae in the presence/absence of predaceous C. carnea larvae (ng/g dry weight). [file Table2.PDF]

Supplemental Table S2 Targeted-analysis of free amino acids

A. Mean (± SD) concentration of free amino acids in the leaves of tomato *S. lycopersicum* `Ailsa Craig´ after 4 weeks of treatment with spider mites *T. urticae* and/or aphids *M. persicae* in the presence/absence of predaceous C. carnea larvae (ng/g dry weight)

LEAF (N=7)

Treatments		Urea *	Asp *	Thr *	Ser	Asn *	Glu	Gln *	Gly	Ala *	Cit *	Val *	Met	Ile *	Leu
Control		**19.04** ab	**30.86** a	**6.87** a	**8.49** a	**137.59** ac	**13.04** a	**13.55** ab	**3.80** a	**6.11** a	**1.31** ab	**8.55** a	**0.49** a	**3.63** a	**2.77** a
	±	6.94	9.34	2.42	2.88	31.18	10.43	12.00	1.35	1.78	2.58	5.17	0.41	1.29	0.86
MeJA		**11.29** ab	**19.04** bc	**2.41** c	**6.44** a	**58.98** b	**8.32** a	**13.96** ab	**2.47** a	**13.31** b	**2.39** ab	**3.82** abc	**0.38** a	**2.91** ab	**2.20** a
	±	6.34	5.87	0.98	2.06	13.47	3.96	18.33	0.65	3.42	1.73	2.83	0.59	0.82	0.89
Predator		**15.89** ab	**17.83** b	**4.03** bc	**5.77** a	**64.68** b	**7.13** a	**4.12** ab	**2.65** a	**11.70** bcd	**0.53** b	**3.45** bc	**0.31** a	**2.34** b	**1.95** a
	±	10.39	2.51	0.40	2.00	4.69	2.60	5.25	0.64	1.97	0.70	2.06	0.29	0.40	0.38
TU		**20.53** ab	**29.59** ac	**6.12** ab	**8.52** a	**106.46** cd	**11.43** a	**1.93** a	**3.33** a	**8.05** ad	**0.82** ab	**8.39** ab	**0.39** a	**2.83** ab	**2.25** a
	±	7.22	10.33	1.11	2.33	11.16	4.59	3.36	1.12	2.25	0.87	3.94	0.22	0.34	0.32
TU-Predator		**8.38** a	**19.04** bc	**4.31** bc	**6.04** a	**73.13** b	**6.96** a	**22.39** b	**3.42** a	**13.07** b	**3.11** a	**2.13** c	**0.19** a	**2.47** ab	**1.88** a
	±	3.35	4.36	0.80	1.44	14.02	1.58	16.51	0.72	4.80	1.93	3.00	0.20	0.52	0.27
MP		**19.95** ab	**29.67** ac	**5.72** ab	**6.61** a	**121.31** ad	**11.24** a	**13.27** ab	**2.96** a	**6.10** a	**0.29** b	**7.31** abc	**0.35** a	**3.12** ab	**2.39** a
	±	8.00	5.76	0.53	1.57	18.12	2.71	8.00	1.15	1.24	0.53	1.77	0.05	0.45	0.40
MP-Predator		**28.40** b	**23.44** abc	**5.00** abc	**5.54** a	**78.21** b	**8.43** a	**18.04** ab	**3.03** a	**12.11** bd	**0.67** b	**4.78** abc	**0.83** a	**2.46** ab	**1.93** a
	±	21.86	5.50	1.18	1.62	16.37	2.48	8.29	0.65	2.34	1.34	2.00	1.20	0.74	0.49
TUMP		**20.72** ab	**27.90** abc	**6.48** abc	**9.12** a	**119.85** ad	**12.43** a	**7.31** ab	**3.85** a	**7.38** ac	**0.44** b	**6.97** abc	**0.39** a	**3.02** ab	**2.51** a
	±	8.38	6.00	1.74	2.03	14.77	5.67	9.85	1.02	2.01	0.43	0.98	0.19	0.33	0.34
TUMP-Predator		**17.03** ab	**21.44** abc	**4.87** abc	**6.56** a	**77.85** b	**11.36** a	**18.94** ab	**3.20** a	**6.16** a	**1.63** ab	**5.90** abc	**0.41** a	**2.56** ab	**1.90** a
	±	4.44	7.85	1.28	1.69	32.55	13.59	14.19	1.05	1.46	0.51	2.45	0.50	0.63	0.46

Treatments		Tyr	Phe *	H-Cystine *	GABA *	His	Trp *	Hyl	Orn *	Lys *	Arg *	Hyp *	Pro	TOTAL in leaf *
Control		**3.80** a	**10.35** a	**3.64** a	**23.43** a	**1.38** a	**28.10** a	**1.54** a	**1.09** ab	**3.81** a	**1.78** a	**124.60** ad	**8.51** a	**468.13** a
	±	3.12	3.37	0.72	7.36	1.06	9.19	0.99	0.47	2.00	1.27	38.66	3.29	112.61
MeJA		**1.99** a	**4.73** b	**2.29** abc	**11.01** b	**0.81** a	**21.58** ab	**2.08** a	**2.04** a	**2.31** b	**0.64** b	**47.53** be	**5.50** a	**250.43** ce
	±	1.35	2.21	1.05	2.88	0.47	5.81	1.03	1.85	0.53	0.29	28.21	1.20	61.21
Predator		**2.26** a	**6.00** ab	**1.45** b	**14.51** ab	**1.06** a	**17.18** b	**1.19** a	**0.31** b	**2.19** b	**0.87** b	**26.63** b	**6.13** a	**222.12** c
	±	0.86	0.70	0.56	2.73	0.71	2.46	0.74	0.10	0.37	0.21	17.97	1.48	38.26
TU		**2.59** a	**8.48** ab	**3.25** ac	**17.52** ab	**2.39** a	**21.68** ab	**1.45** a	**0.92** ab	**2.97** ab	**1.18** ab	**107.77** ac	**9.01** a	**389.85** ab
	±	0.80	2.28	0.86	9.18	3.57	6.26	0.69	0.18	0.53	0.30	40.02	3.45	62.21
TU-Predator		**2.04** a	**7.25** ab	**2.18** bc	**15.18** ab	**1.56** a	**18.61** b	**1.35** a	**0.96** ab	**2.26** b	**0.90** ab	**66.47** bc	**8.28** a	**293.55** bc
	±	0.83	3.44	0.73	7.51	0.98	3.98	0.62	0.35	0.36	0.26	25.61	5.29	41.98
MP		**2.93** a	**9.18** ab	**3.21** ac	**20.94** ab	**0.77** a	**25.94** ab	**1.25** a	**0.88** b	**3.36** ab	**1.35** ab	**106.21** cd	**7.03** a	**413.33** ad
	±	0.97	1.83	0.32	4.96	0.21	5.06	0.36	0.14	0.65	0.43	17.61	2.98	56.25
MP-Predator		**2.06** a	**6.45** ab	**2.63** abc	**16.67** ab	**1.74** a	**18.01** b	**1.40** a	**0.59** b	**2.27** b	**0.92** ab	**95.09** ace	**7.95** a	**348.64** bde
	±	0.52	1.34	0.85	4.63	0.87	3.97	0.70	0.28	0.28	0.23	25.81	2.36	51.25
TUMP		**2.77** a	**9.45** ab	**2.95** ac	**21.88** ab	**1.63** a	**23.89** b	**1.06** a	**0.98** ab	**3.23** b	**1.20** ab	**97.71** bc	**9.04** a	**404.14** ab
	±	0.83	2.63	0.90	5.78	1.02	2.23	0.64	0.05	0.37	0.34	27.77	2.76	45.15
TUMP-Predator		**1.89** a	**9.55** ab	**3.12** ac	**19.89** a	**1.09** a	**17.61** ab	**1.51** a	**0.93** ab	**2.28** ab	**0.92** ab	**67.33** abc	**7.40** a	**313.34** bcd
	±	0.67	6.95	0.96	6.21	0.67	4.15	0.70	0.22	0.44	0.25	33.50	2.22	86.04

* : $p \leq 0.05$ one-way ANOVA, Tukey´s HSD post hoc test: Different lower cases indicate significant differences between the treatments for one given amino acid

MeJA, elicitation with methyl jasmonate (2.5 mM); TU, *T. urticae;* MP*, M. persicae;* TUMP*, both pests;* Predator*, presence of C. carnea* larvae

B. Mean (± SD) concentration of free amino acids in the fruits of tomato *S. lycopersicum* \`Ailsa Craig´ after 4 weeks of treatment with spider mites *T. urticae* and/or aphids *M. persicae* in the presence/absence of predaceous C. carnea larvae (ng/g dry weight)

FRUIT (N=6)

Treatments		Urea	Asp	Thr	Ser	Asn	Glu	Gln	Gly	Ala *	Cit	Val	Met	Ile	Leu
Control		**362.72** a	**41.07** a	**21.67** a	**52.73** a	**28.27** a	**72.41** a	**2815.22** a	**4.31** a	**35.42** a	**0.76** a	**27.59** a	**1.10** a	**12.58** a	**10.72** a
	±	117.57	12.39	6.35	14.29	16.01	29.29	1541.35	0.67	8.39	0.85	6.55	0.35	4.14	2.97
MeJA		**263.86** a	**30.25** a	**23.40** a	**56.08** a	**48.75** a	**55.83** a	**1894.29** a	**5.45** a	**53.18** bc	**0.30** a	**26.76** a	**1.36** a	**14.88** a	**11.81** a
	±	122.59	14.62	12.45	24.99	27.93	24.29	1025.17	2.45	17.67	0.35	14.84	0.76	7.86	6.53
Predator		**294.87** a	**28.66** a	**21.56** a	**50.15** a	**35.08** a	**52.02** a	**1564.45** a	**4.31** a	**50.41** abc	**0.86** a	**19.63** a	**1.25** a	**13.00** a	**10.98** a
	±	137.94	14.48	7.71	11.97	13.00	15.32	872.10	1.59	13.68	2.04	12.16	0.69	5.40	4.92
TU		**234.32** a	**28.19** a	**21.35** a	**53.17** a	**32.76** a	**48.60** a	**1328.33** a	**5.18** a	**50.88** abc	**0.38** a	**24.29** a	**0.92** a	**12.43** a	**9.31** a
	±	108.08	5.61	3.98	12.83	18.17	15.16	312.50	1.49	5.10	0.39	5.67	0.23	2.26	0.96
TU-Predator		**345.77** a	**39.38** a	**24.68** a	**61.41** a	**42.62** a	**60.01** a	**2666.38** a	**5.23** a	**39.97** ab	**0.00** a	**33.13** a	**1.12** a	**14.33** a	**10.00** a
	±	92.47	5.81	7.02	15.27	17.19	11.87	1076.63	1.73	4.38	0.00	7.49	0.32	4.72	3.04
MP		**372.52** a	**41.40** a	**22.22** a	**52.57** a	**41.09** a	**65.27** a	**2609.93** a	**4.80** a	**37.52** ab	**0.35** a	**32.01** a	**1.50** a	**12.67** a	**12.52** a
	±	100.55	8.52	4.47	14.36	14.76	6.09	847.29	0.43	3.13	0.41	4.20	0.53	1.74	3.46
MP-Predator		**294.48** a	**36.75** a	**26.03** a	**60.40** a	**48.19** a	**49.16** a	**1602.21** a	**5.53** a	**58.24** c	**0.11** a	**30.24** a	**1.24** a	**14.96** a	**11.90** a
	±	45.11	4.02	1.53	3.27	9.63	6.83	267.43	0.47	5.39	0.28	2.79	0.51	1.49	2.76
TUMP		**397.11** a	**38.54** a	**23.40** a	**59.68** a	**41.32** a	**67.97** a	**2665.33** a	**5.41** a	**39.47** ab	**0.16** a	**30.47** a	**0.71** a	**12.17** a	**9.46** a
	±	79.18	4.68	2.68	8.72	8.68	9.53	619.86	0.69	4.61	0.39	3.79	0.42	1.95	2.06
TUMP-Predator		**238.53** a	**24.65** a	**23.22** a	**57.52** a	**43.75** a	**53.72** a	**1743.48** a	**4.85** a	**54.49** bc	**2.52** a	**24.47** a	**0.91** a	**13.56** a	**9.84** a
	±	92.65	16.15	9.36	20.11	28.47	20.69	735.51	1.47	10.98	3.86	8.39	0.49	4.79	3.01

Treatments		Tyr	Phe	H-Cystine *	GABA *	His	Trp	Hyl	Orn	Lys	Arg	Hyp *	Pro	TOTAL in fruit
Control		**25.87** a	**18.86** a	**1.39** a	**349.28** acd	**9.28** a	**4.29** a	**7.11** a	**0.00** a	**10.73** a	**11.31** a	**56.92** a	**3.84** a	**3985.45**
	±	9.87	7.46	1.53	62.60	4.21	1.90	2.44	0.00	2.68	2.93	26.61	1.45	1774.21
MeJA		**22.88** a	**22.38** a	**1.38** ab	**256.90** bc	**10.62** a	**5.92** a	**6.56** a	**0.00** a	**10.71** a	**11.34** a	**5.62** b	**2.60** a	**2843.12**
	±	9.03	10.02	0.34	64.30	4.70	2.15	3.28	0.00	5.09	6.20	1.61	1.19	1384.83
Predator		**22.21** a	**18.14** a	**2.20** ab	**261.56** bc	**7.59** a	**5.35** a	**6.49** a	**0.00** a	**10.46** a	**10.85** a	**6.24** b	**2.30** a	**2500.60**
	±	6.87	6.36	0.59	61.24	2.09	1.16	2.03	0.00	3.10	3.41	1.40	0.83	1153.73
TU		**19.64** a	**18.22** a	**1.97** ab	**232.66** bd	**9.79** a	**3.91** a	**6.74** a	**0.00** a	**9.75** a	**10.81** a	**6.05** b	**1.96** a	**2171.61**
	±	4.48	2.32	0.45	43.73	1.55	1.12	0.85	0.00	1.16	1.60	1.49	0.49	493.73
TU-Predator		**23.41** a	**19.21** a	**1.93** ab	**290.08** abc	**10.84** a	**4.83** a	**7.61** a	**0.00** a	**10.76** a	**12.05** a	**50.90** a	**2.10** a	**3777.76**
	±	6.99	6.01	0.08	49.79	3.54	0.83	2.18	0.00	3.29	4.05	13.10	0.28	1274.75
MP		**28.03** a	**20.04** a	**2.29** ab	**383.02** a	**9.64** a	**4.83** a	**6.75** a	**0.00** a	**11.61** a	**12.51** a	**62.36** a	**3.22** a	**3850.67**
	±	5.14	4.02	0.32	82.65	3.07	1.03	1.92	0.00	2.43	2.54	8.99	1.16	1011.47
MP-Predator		**23.29** a	**19.91** a	**1.42** ab	**258.79** bc	**9.16** a	**5.65** a	**7.89** a	**0.00** a	**11.72** a	**13.16** a	**4.32** b	**2.63** a	**2597.37**
	±	3.16	2.60	0.29	56.85	0.81	1.64	1.59	0.00	1.65	1.59	2.39	0.89	313.54
TUMP		**25.45** a	**16.10** a	**1.91** ab	**359.37** ac	**10.00** a	**4.38** a	**6.60** a	**0.00** a	**10.39** a	**11.52** a	**54.93** a	**2.34** a	**2607.96**
	±	7.49	2.11	0.19	90.51	1.62	0.95	1.42	0.00	1.57	2.70	10.49	0.57	876.03
TUMP-Predator		**19.51** a	**18.78** a	**2.76** b	**214.57** b	**9.40** a	**5.53** a	**7.12** a	**0.22** a	**10.24** a	**12.79** a	**5.71** b	**5.80** a	**3894.19**
	±	4.53	5.07	1.24	30.32	2.99	1.11	2.87	0.55	3.29	5.54	0.79	6.18	745.02

* : $p \leq 0.05$ one-way ANOVA, Tukey´s HSD post hoc test: Different lower cases indicate significant differences between the treatments for one given amino acid
MeJA, elicitation with methyl jasmonate (2.5 mM); TU, *T. urticae;* MP, *M. persicae;* TUMP, *both pests;* Predator, *presence of C. carnea* larvae
