# Supplementary material for: Metabolite Profiling Reveals a Specific Response in Tomato to Predaceous Chrysoperla carnea Larvae and Herbivore(s)-Predator Interactions with the Generalist Pests Tetranychus urticae and Myzus persicae
Source: Front Plant Sci. 2016 Aug 25;7:1256. doi: 10.3389/fpls.2016.01256 (PMC4997045; doi:10.3389/fpls.2016.01256)
Supplement: Supplementary Table S3 — Mean (±SD) peak area of the most abundant volatiles emitted by tomato S. lycopersicum “Ailsa Craig” leaves after four weeks of treatment with spider mites T. urticae and/or aphids M. persicae in the presence/absence of predaceous C. carnea larvae. [file Table3.PDF]

<u>Supplemental Table S3</u> **Mean (± SD) peak area of the most abundant volatiles emitted by tomato *S. lycopersicum* \`Ailsa Craig´ leaves after 4 weeks of treatment with spider mites *T. urticae* and/or aphids *M. persicae* in the presence/absence of predaceous *C. carnea* larvae**

**Compound**				**Treatments (mean peak area 10^6)**								
**Epidermis**	**Class**	**Name** (*confirmed with reference substrance)	**RT (min)**	**Control**	**MeJA**	**Predator**	**TU**	**TU-Predator**	**MP**	**MP-Predator**	**TUMP**	**TUMP-Predator**
adaxial	monoterpenes	*α*-phellandrene*	17.5	269 ± 195 a	383 ± 217 a	324 ± 231 a	557 ± 406 a	496 ± 180 a	527 ± 394 a	731 ± 604 a	242 ± 178 a	555 ± 97 a
		β-phellandrene*	19.5	4038 ± 2040 a	5587 ± 3006 a	4464 ± 2694 a	6647 ± 3951 a	6676 ± 1852 a	6283 ± 3390 a	8252 ± 5883 a	3846 ± 2358 a	5471 ± 2036 a
	sesquiterpenes	*β*-caryophyllene*	35.1	162.5 ± 89.4 a	300.9 ± 271.3 a	125.1 ± 104.4 a	187.3 ± 155.7 a	208.3 ± 150.5 a	153.2 ± 80.1 a	274.4 ± 199.5 a	116.1 ± 115.0 a	210.4 ± 91.3 a
abaxial	monoterpenes	*α*-phellandrene*	17.5	140 ± 77 a	86 ± 37 a	170 ± 109 a	233 ± 219 a	232 ± 123 a	229 ± 214 a	267 ± 339 a	157 ± 129 a	107 ± 78 a
		β-phellandrene*	19.5	2515 ± 1328 a	1611 ± 510.7 a	12758.0 ± 2225	3237 ± 2698 a	3446 ± 1950 a	3504 ± 2736.5 a	3829.0 ± 4318 a	2664 ± 2008 a	1107 ± 660 a
	sesquiterpenes	*β*-caryophyllene*	35.1	117.5 ± 75.1 a	52.3 ± 26.5 a	57.1 ± 33.2 a	144.3 ± 132.9 a	103.7 ± 95.9 a	133.8 ± 107.9 a	134.9 ± 201.0 a	93.6 ± 75.3 a	7.5 ± 4.9 a

abbreviations: MeJA, elicitation with methyl jasmonate (2.5 mM); MP, aphids *M. persicae*; TU, spider mites *T. urticae;* Predator, green lacewing larvae *C. carnea*

RT, retention time; different lower case letters indicate significant differences between the treatment ($p \leq 0.05$, one way ANOVA, Tukey' HSD post-hoc test, N = 4)
